# Using Large Language Model to Optimize Protein Purification: Insights from Protein Structure Literature Associated with Protein Data Bank

**DOI:** 10.1002/advs.202413689

**Published:** 2025-02-20

**Authors:** Zhuojian Chen, J. Sivaraman

**Affiliations:** ^1^ Department of Biological Sciences National University of Singapore 14 Science Drive 4 Singapore 117543 Singapore

**Keywords:** articles, database, information extraction, proteins, purification, strategies

## Abstract

Obtaining pure and homogeneous protein samples is vital for protein biology studies, yet optimizing protein expression and purification methods can be time‐consuming because of variations in factors like expression conditions, buffer components, and fusion tags. With over 81 000 Protein Data Bank (PDB)‐associated articles as of October 2024, manual extraction of relevant methods is impractical. To streamline this process, an automated tool is developed by incorporating a large language model (LLM) to extract and classify key data from these articles. The information extraction accuracy is enhanced by a 2‐step‐LLM and a 3‐step‐prompt. The key findings include: 1) Tris buffer is used in 49.2% of cases, followed by 4‐(2‐hydroxyethyl)‐1‐piperazineethanesulfonic acid (HEPES) and phosphate buffers. 2) Polyhistidine tags dominate at 82.5%, followed by glutathione S‐transferase (GST) and maltose‐binding protein (MBP) tags. 3) *E. coli* expression is done at 16–20 °C, with induction period favoring 12–16 h (69.0%) over 3–6 h (14.3%). The statistical analyses highlight the correlation between protein properties and purification strategies. This tool is validated through two case studies: method bias for membrane protein purification, and crosslinker/detergent preferences for Cryo‐Electron Microscopy sample preparation. These findings provide a valuable resource for designing protein expression and purification experiments.

## Graphical abstract

An automated tool incorporating a large language model (LLM) streamlines the extraction and classification of protein expression and purification methods from 64 909 PDB‐associated articles. By identifying trends in buffer components, fusion tags, expression conditions, and crosslinker/detergent preferences, the tool provides valuable insights into the correlation between protein properties and purification strategies.

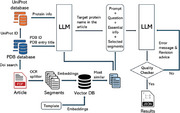

## 1 Introduction

The success of structural studies, drug discovery, and biophysical and biochemical assays relies on the isolation and purification of target proteins. The purification process is necessary to separate proteins from other proteins and non‐protein components in a mixture, thus ultimately isolating the desired protein from contaminants.^[^ ^1^ ^]^ To accurately examine the structural and functional properties of a protein of interest, it must be isolated from other cellular components to prevent interference or interactions.^[^ ^2^ ^]^ Typically, however, such isolation means that proteins of interest are also separated from potential stabilizing factors, and this can lead to a loss of function and reduced stability.^[^ ^3^ ^]^ To combat this, protein samples can be stabilized by adjusting the pH or salt concentration of the buffer systems/combinations, or by adding specific additives such as glycerol or detergents. Indeed, identifying the optimal stabilizing conditions for a protein of interest not only enhances target protein purification but also improves its stability for subsequent structural and functional studies.^[^ ^4^ ^]^ Furthermore, while it is also required for isolation, the introduction of a fusion tag can positively influence the biochemical properties of the recombinant construct, enhance its expression, solubility, refolding efficiency, and prevent proteolysis.^[^ ^5^ ^]^ Despite having all this knowledge, choosing an effective expression and purification strategy for an unknown protein typically relies on time‐consuming empirical methods and trial‐and‐error experiments.

Recent advances in natural language processing (NLP) have led to the development of powerful large language models (LLMs) such as the GPT (Generative Pre‐trained Transformer) series, including ChatGPT^[^ ^6^ ^]^ and LLaMA 3.^[^ ^7^ ^]^ These models, pre‐trained on extensive text data, have demonstrated exceptional performance across a wide range of NLP tasks, including language translation, text summarization, and question answering. Text embedding, a longstanding topic in NLP and information retrieval, represents texts with latent semantic vectors, supporting various applications such as web search, question answering, and retrieval‐augmented language modeling.^[^ ^8^ ^]^ The application of LLMs and text‐embedding models significantly reduces the time and labor required for extracting and analyzing relevant information from article databases.

In this study, we propose an LLM‐based tool for article information extraction, adaptable to various styles of articles related to protein studies. We applied this tool to extract protein expression and purification strategies from 64 909 articles that contributed entries to the RCSB Protein Data Bank. The database and literature extraction analysis reported here is the largest of its kind hitherto reported. Moreover, through statistical analyses, we found correlations between bias in the empirical choices of protein expression and purification strategies and the properties of target proteins. These analyses serve as guidelines for reducing the trial‐and‐error process associated with initiating studies on new and uncharacterized proteins with unknown structures.

## 2 Results

### 2.1 Efficient Article Information Extraction Tool

**Figure 1:**
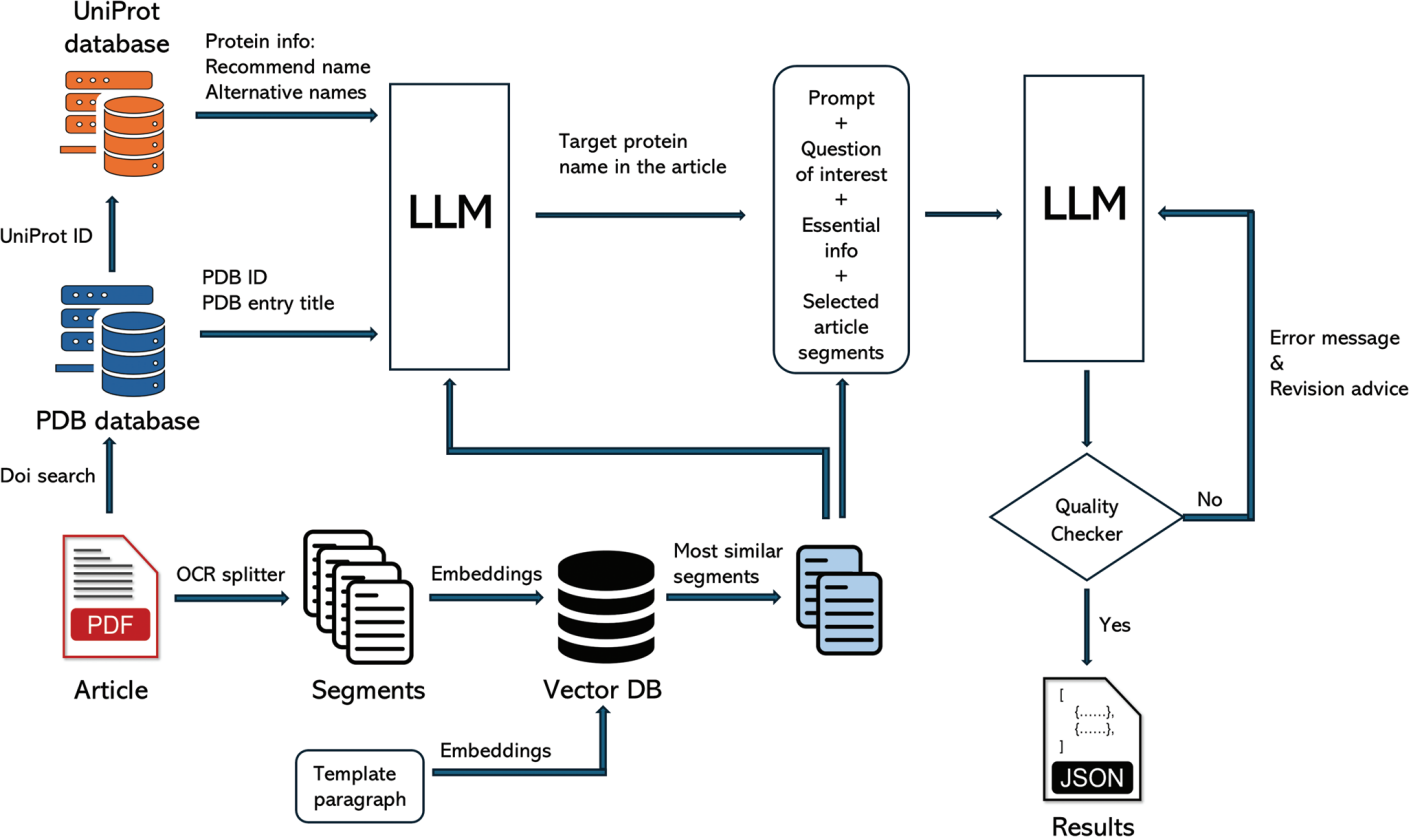
Workflow of the Efficient Article Information Extraction Tool. Article section embeddings were calculated using the bge‐large‐en‐v1.5 model. LLM stands for Large Language Model. We used LLaMA 3 70B Instruct for information extraction and database construction.

An overview of the Article Information Extraction Tool is shown in **Figure** **1**. To begin, PDF files of the articles were preprocessed using Optical Character Recognition (OCR), which is compatible with either text‐based or scanned PDF formats. The tool then segmented each article into chunks containing no more than 2500 characters, with chunking based on subtitles. Given that the content of each section divided by subheadings is relatively independent in biological articles, chunking retained the integrity of each section of an article and thus improved the performance of the LLM in the downstream information extraction.

We built our protein purification database from 64 909 articles, which is ≈ 80% of the total number, that could be downloaded and are associated with PDB protein 3D structure entries. Initial analyses showed that 83.9% of these articles contained 10 000 to 40 000 LLM tokens. If the article, together with the target question, was simply fed into the LLM for information extraction, the length of the input would have exceeded the maximum tokens of many LLMs. Even though we can use LLMs with long context lengths, any unnecessary context would not only have increased the model hallucination but also caused some of the points related to answers in the articles to be ignored. Indeed, the answers to our questions of interest are highly likely to be found within a few paragraphs of an article. For example, buffer details for isolating and purifying target proteins are most likely found under the protein expression and purification subsection of the methods. To effectively and accurately locate the segments related to the question of interest, we built a vector database of all articles using the bge‐large‐en‐v1.5 model to calculate the embedding of each chunk.^[^ ^9^ ^]^ The cosine similarity between the embedding of a template paragraph based on the question of interest and the embeddings in the article database is positively correlated with the probability that the information would be found within the corresponding segment.

In biology articles, target protein names can appear as full, recommended names, alternative names, or even abbreviations. To accurately identify targets, we constructed a dictionary mapping protein names and abbreviations using information from the PDB and UniProt databases. This mapping information was further processed by the LLM to determine the correct target protein names presented in the article. We utilized LLaMA 3 70B Instruct for this task. Subsequently, the identified protein names, questions of interest, and selected segments of the article were used to construct prompts for the LLM. The output generated by the LLM was then examined by a quality checker program to enhance the accuracy of data extraction.

### 2.2 2‐Step‐LLM Process and 3‐Step‐Prompt Significantly Decrease the Error Rate of Information Extraction

Correct information extraction is crucial; incorrect labels, for example, can severely compromise subsequent statistical analyses of protein purification strategies. We found that integrating segment ranking based on embedding similarity, a 2‐step‐LLM process, and a 3‐step‐prompt provided us with the lowest observed error rate (0.67%) for extracting details of buffer components (Figure S1, Supporting Information). The typical range of tokens for most articles is between 10 000 and 40 000. When combined with a 1300‐token prompt, the input frequently exceeded the maximum token limit for many LLMs, such as LLaMA 3 70B Instruct. Additionally, structural biology articles typically describe buffer details in the materials and methods sections. Thus, the embedding model is able to effectively identify relevant paragraphs containing the target information, thereby excluding unnecessary parts of the articles and reducing confusion for the LLM.

Many articles used acronyms, gene names, or other symbolic names instead of recommended names in the method sections, leading to incorrect information extraction by the LLM. An additional preprocessing step was thus deemed necessary and involved an analysis of corresponding PDB and UniProt data to identify the correct names of target proteins mentioned in the articles. This preprocessing step, as shown in Figure S1 (Supporting Information), significantly reduced the error rate. The design of the prompt structure also significantly influenced the extraction outcome. Compared with directly instructing the LLM to extract and classify buffers or chemicals, initiating the response with the copied sentences related to buffer details effectively reduced the frequency of model hallucinations. This approach was particularly beneficial when mapping buffer relationships in articles that examined various protein types.

### 2.3 Statistics of the Database of Purification Strategies

Of the 81 404 articles contributing entries to the RCSB Protein Data Bank, a total of 64 909 (≈80%) articles were processed and incorporated into this database (Figure S2A, Supporting Information). Among these, 42 732 articles were mapped to UniProt IDs from the Swiss‐Prot and TrEMBL protein databases, resulting in 30 934 proteins with unique UniProt IDs within our database. Figure S2B (Supporting Information) illustrates the distribution of methods used to determine the protein 3D structures in the articles analyzed by our Efficient Article Information Extraction Tool.

The 3D structures included 81 821 unique PDB entries from X‐ray diffraction, 5690 entries from solution nuclear magnetic resonance (NMR), and 5153 entries from electron microscopy. Each entry contains comprehensive information, including the fused tag, the recombinant protein expression system, the culture conditions, the induction strategy, and details of the buffers used in the purification process (such as pH, concentration, components, and functions). Moreover, we mapped the proteins to the UniProt database and integrated the recommended name of the protein, the UniProt ID, the PDB ID, the DOI of the article, and the protein sequence into each entry. However, due to incomplete information in the original articles, not all entries in our extracted database contain all of these labels.

### 2.4 Significant Variability in the Frequency Distribution of Buffer Usage

**Figure 2:**
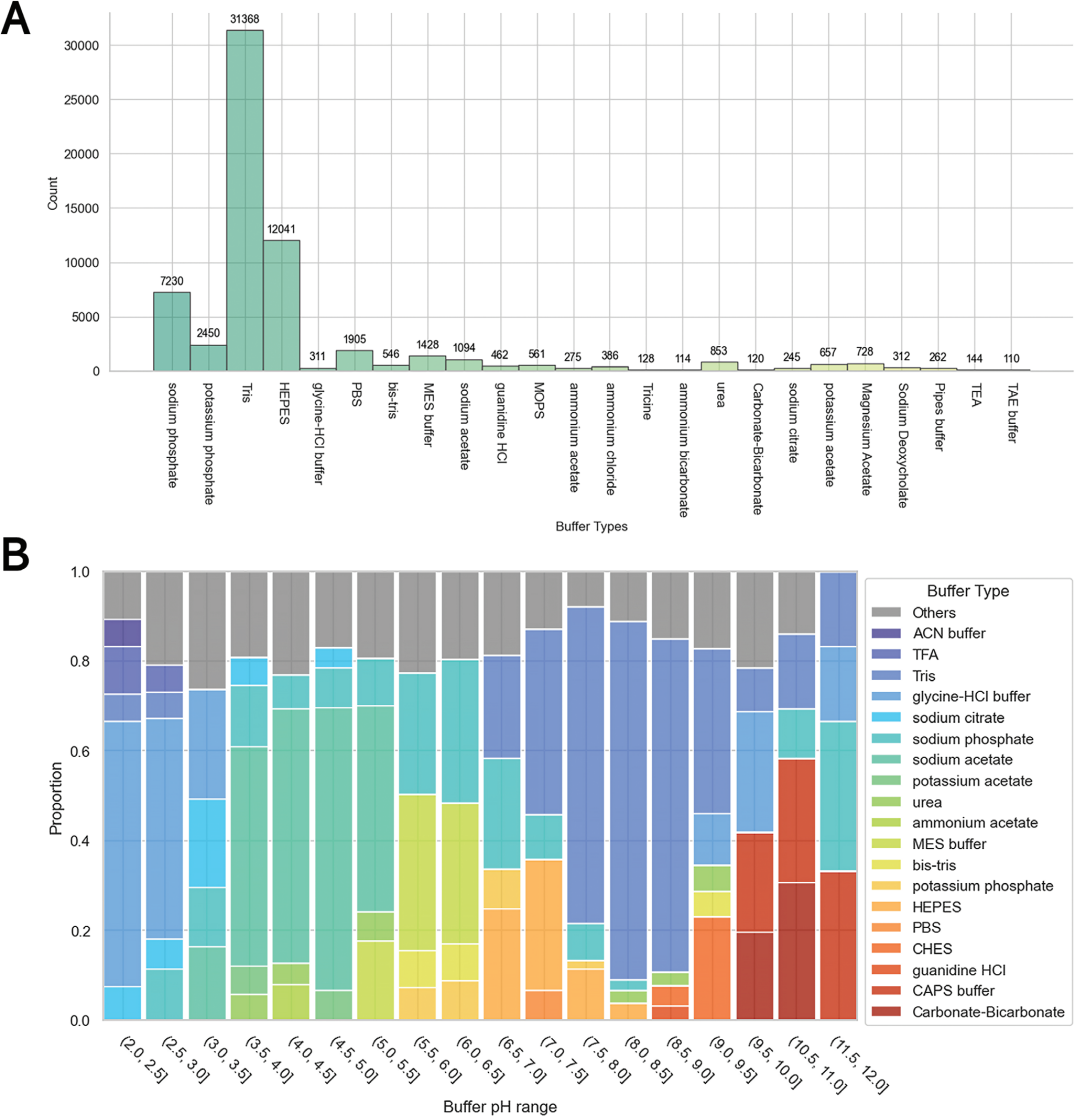
Statistics of Buffer Types in Our Database. A) Frequency of buffer usage cases. Each unique buffer type utilized in the purification of a protein, identified by a unique UniProt ID, is counted as one case. B) Distribution of buffer usage across different pH ranges. The sixth‐most frequent buffers and all subsequent buffers are aggregated into the category “Others”. The total number of records is 63 729.

Buffers are essential in protein purification protocols/experiments as they help solutions resist changes in pH when small quantities of acid or base are added. Protein solubility is influenced by several factors, with buffer pH playing a critical role. Solubility is typically lowest near the isoelectric point of the protein.^[^ ^10^ ^]^ The pH of a buffer can affect the charge, shape, and hydrophobicity of proteins, thus influencing their solubility. Our database extraction analysis demonstrated significant variability in the buffers used (**Figure** **2**A). Tris buffer was the most frequently used buffer across the studies, appearing in 31 368 cases and accounting for 49.2% of all buffer types. 4‐(2‐hydroxyethyl)‐1‐piperazineethanesulfonic acid (HEPES buffer was used in 12 041 cases, whereas phosphate buffers appeared in 9680 cases, making them the second‐ and third‐most prevalent buffer types, respectively. The remaining 20 buffer types were used in fewer than 2000 cases.

Each buffer operates efficiently within a specific pH range. Figure 2B illustrates the distribution of buffer selections across different pH ranges. Glycine‐HCl buffer was most commonly used at low pH values (≤ pH 3.5), whereas acetate buffers were frequently used in the pH range of 3.5 to 6.5. Phosphate and HEPES buffers were predominantly used in the pH range of 6.5 to 7.5. Tris buffer was the most popular choice for neutral to slightly alkaline environments (pH 7.0 to 9.5). For highly alkaline environments (≥ pH 9.5), carbonate‐bicarbonate buffer and CAPS (N‐cyclohexyl‐3‐aminopropanesulfonic acid) buffer were preferred. Buffers that are frequently used are likely to be compatible with a wide range of proteins. Therefore, it is prudent to start with commonly used buffers when purifying an unknown and uncharacterized protein.

### 2.5 Correlation of Buffer Components and Protein Annotations

Generally, protein purification buffers include salts like sodium chloride (NaCl), to maintain protein solubility and mimic physiological conditions. Reducing agents may also be included to prevent disulfide bond formation due to oxidization of cysteine amino acids in proteins. Detergents, such as Tween 20 or Triton X‐100, can also be incorporated into a protein purification buffer to stabilize membrane proteins in solution. Finally, numerous additives can help to increase the effectiveness of a protein purification experiment; for example, glycerol and PEG (Polyethylene glycol) can prevent aggregation and stabilize proteins, whereas some ionic compounds like sulfates, amino acids, and citrates, can shield ionic interactions and solubilize proteins.

From the 64 909 analyzed articles, we identified 72 common buffer components used in the purification of over 100 different types of proteins. UniProt provides comprehensive protein annotation information,^[^ ^11^ ^]^ which we integrated with our extraction results. The heatmap presented in Figure S3 (Supporting Information) highlights associations between buffer components and the protein annotations. Upon inspection, it is evident that specific chemicals are strongly associated with certain annotations. For example, diverse usage patterns of detergents are associated with the molecular function category. Detergents such as cholesteryl hemisuccinate (CHS, 21‐fold), n‐dodecyl‐β‐D‐maltoside (DDM, 6.1‐fold), digitonin (22‐fold), and lauryl maltose neopentyl glycol (LMNG, 9‐fold) are associated with proteins having ion channel function, whereas the same four detergents are largely negatively correlated with protein purification of kinases. Instead, Nonidet P‐40 (NP‐40, 2.2‐fold), Triton X‐100 (1.8‐fold), and Tween 20 (1.7‐fold) are more frequently used in kinase purification. Besides, phosphatase inhibitors, including sodium fluoride (NaF, 5.7‐fold) and sodium orthovanadate (Na_3_VO_4_, 10.4‐fold), are frequently associated with purifying kinases, which is not surprising as such inhibitors are essential to preserve the kinase phosphorylation state and activity.

**Figure 3:**
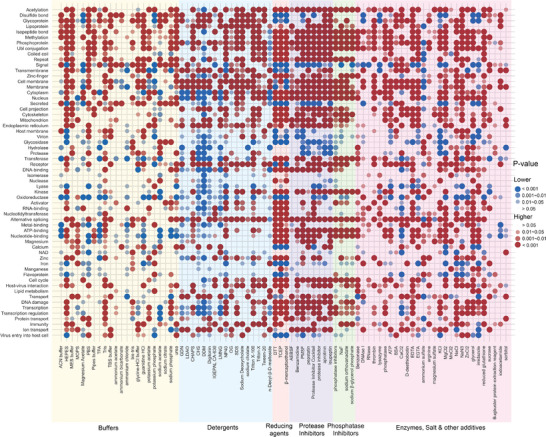
Two Proportion Z‐Tests of Buffer Components Between Proteins with and without a Specific UniProt Annotation. This dataset contains 22 833 unique UniProt IDs. To minimize the impact of outliers, only annotations present in over 500 unique entries in our database are included in the test. *P*‐values were calculated using Two Proportion Z‐Tests. The blue dots indicate cases where the frequency of a specific chemical usage was significantly lower in the protein group with a particular annotation compared to the group without that annotation, while the red dots indicate the opposite cases where the frequency was significantly higher.

The two proportion Z‐Tests (**Figure** **3**) on the entries in our database provided us with another perspective regarding the associations between protein annotations and buffer components. Proteins with different annotations showed varied preferences for buffer types: over 10 types of buffers were used in significantly high proportions for the purification of proteins with annotations such as “acetylation”, “isopeptide bond”, and “Ubl conjugation”. In contrast, for proteins with annotations such as “oxidoreductase”, “ATP‐binding”, and “nucleotide‐binding”, most of the buffer types have significantly lower usage in their respective purification steps, which makes the buffers of higher usage in these proteins would be considerably important in practice; for instance, 3‐(N‐morpholino)propanesulfonic acid (MOPS) and phosphate buffers were highly associated with annotations of “oxidoreductase”. Additionally, proteins with most of the annotations listed in Figure 3 were linked with a higher usage of reducing agents such as dithiothreitol (DTT), tris(2‐carboxyethyl) phosphine (TCEP), and β‐Mercaptoethanol. However, we also identified some exceptions, such as “disulfide bonds”, “glycoproteins”, “signaling proteins”, “virions”, which were linked with non‐reducing environments in the protein purification process. Furthermore, significantly higher frequencies of numerous non‐ionic detergents were noticed in the purification of transmembrane proteins, with the exception of NP‐40 that might be attributed to its relatively harsh properties and large micelle size causing suboptimal performance in maintaining protein stability and functionality. NP‐40 being less frequently used underscores the importance of carefully optimizing the choice of detergent for each transmembrane protein to ensure preservation of its structural and functional integrity during purification. In summary, the relationship between protein properties and the usage frequency of various buffer components highlights the necessity of selecting appropriate buffer conditions to enhance the efficiency and specificity of the protein purification processes. This detailed understanding aids the rational design of buffer systems tailored to the biochemical and structural needs of different protein types, thereby facilitating improved purification outcomes.

### 2.6 Correlation of Fusion Tags and Protein Properties

**Figure 4:**
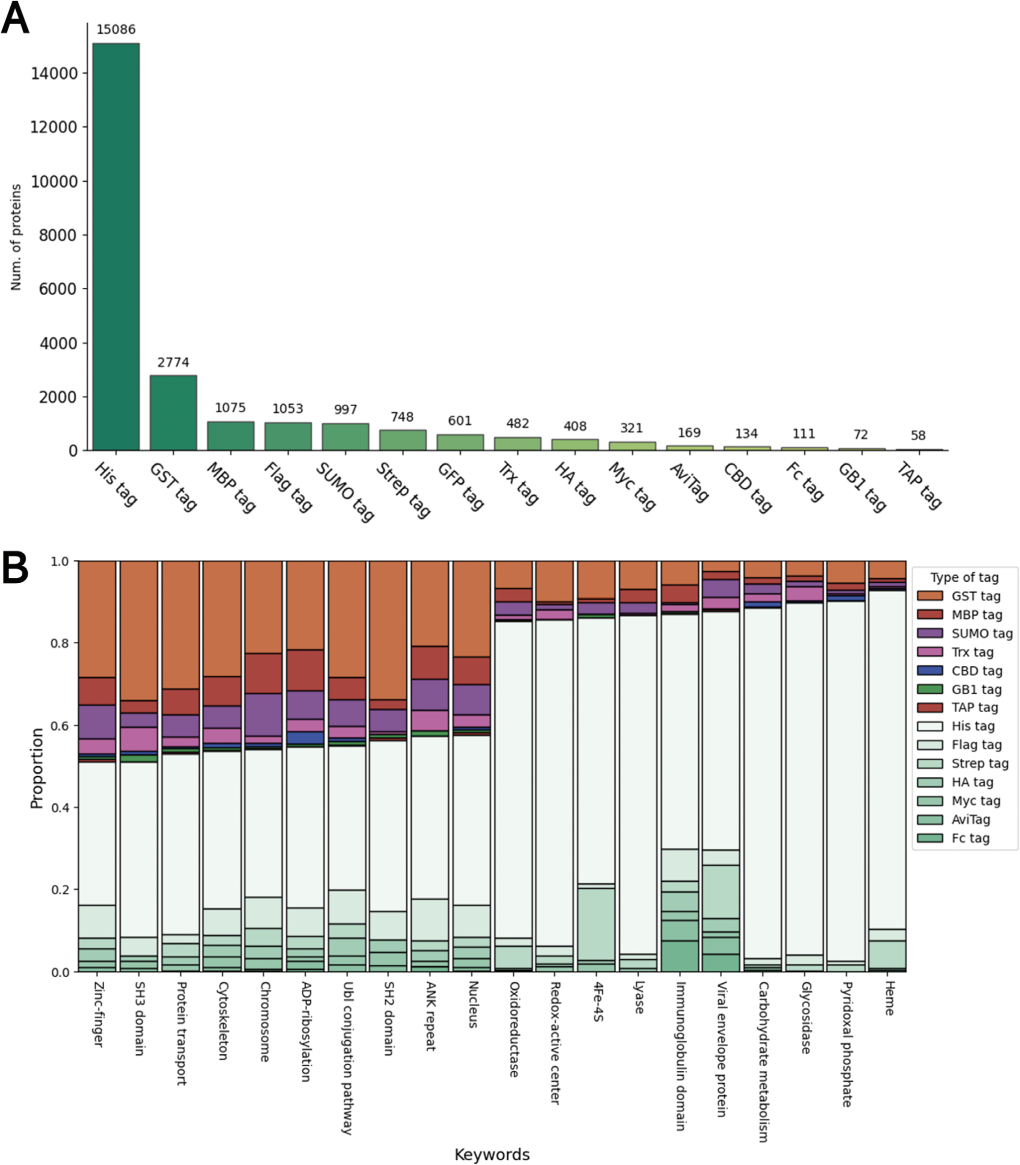
Statistics of Fusion Tag Usage in Our Database: A) Overall fusion tag usage for recombinant proteins with 18 288 unique UniProt IDs. Note that a single recombinant protein, corresponding to a unique UniProt ID, may have records of multiple types of fusion tags. B) Top 10 and bottom 10 protein properties associated with the use of large fusion tags. GST tag, MBP tag, SUMO tag, thioredoxin‐1 (Trx) tag, cellulose‐binding domain (CBD) tag, B1 domain of Streptococcal protein G (GB1) tag, and tandem affinity purification (TAP) tag were classified as large tags, while His tag, FLAG tag, Strep tag, hemagglutinin (HA) tag, Myc tag, AviTag and Fc tag were classified as small tags. If a recombinant protein contains both large and small fusion tags, it is classified under the type of the large fusion tag only.

Using our Article Information Extraction Tool, we identified fusion tags for 12 773 unique UniProt entries in the Swiss‐Prot database and 5515 entries in the TrEMBL protein database. The polyhistidine tag was identified as the most frequently used fusion tag, found in 82.5% of the recombinant proteins in the database (**Figure** **4**A). The other three most popular large fusion tags were glutathione S‐transferase (GST) tag, maltose‐binding protein (MBP) tag, and small ubiquitin‐related modifier (SUMO) tag. These tags enhance the solubility of recombinant proteins, thereby increasing purification efficiency.^[^ ^12^ ^]^ A single entry may involve more than one type of tag due to the use of tandem tag combinations, different types of tags at the N‐ and C‐termini, and compatibility of the protein with multiple types of tags.

Producing high‐quality protein for functional assays and structural studies, such as NMR spectroscopy, X‐ray crystallography, and cryo‐electron microscopy, requires multi‐milligram quantities of protein of high purity. A significant portion of recombinant proteins exhibit low expression levels or poor solubility, particularly when using *E. coli* expression systems. Depending on the properties of the proteins, incorporating fusion tags can mitigate some of these challenges by increasing yield, enhancing folding, and streamlining purification processes.^[^ ^13^ ^]^ Figure 4B illustrates the diversity in preference for recombinant proteins fused with large tags. For example, nearly 50% of recombinant proteins with Zinc‐finger or SRC Homology 3 (SH3) domains were expressed with large fusion tags, indicating that these proteins are more likely to be compatible with or require large tags for expression and solubilization. In contrast, only 9% of recombinant proteins containing at least one heme were expressed with large tags. Multidomain fusion proteins joined by a flexible linker may be less likely to form well‐ordered, diffraction‐quality crystals or may be too large for NMR studies. Strategies requiring tag removal introduce additional challenges including optimization of cleavage conditions and add costs for proteases. Therefore, starting with a small fusion tag, such as a polyhistidine tag, is advisable for proteins with annotations that are less frequently associated with the use of large fusion tags. On the contrary, if the target protein has annotations associated with a large fusion tag, fusion with a GST tag or MBP tag may aid the expression and purification.

### 2.7 Culture Conditions of Recombinant Protein Expression

*E. coli* remains the most widely used system for recombinant protein expression due to its numerous advantages over other expression systems such as yeast, baculovirus, mammalian cells, and cell‐free systems. The key benefits of using *E. coli* include its low cost, ease of use, and ability to scale production efficiently, making it the preferred host for many researchers.^[^ ^14^ ^]^ Furthermore, the flexibility of *E. coli* in accommodating various expression conditions allows for fine‐tuning protein production to optimize yield and solubility, which are critical for downstream applications. Recent studies have demonstrated that altering expression parameters, such as temperature, induction time, and inducer concentration, can significantly enhance production efficiency and solubility of target proteins.^[^ ^15^ ^]^

**Figure 5:**
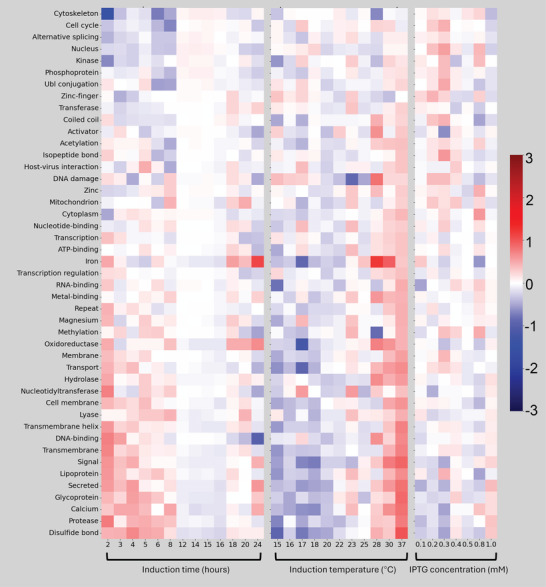
Log fold difference in induction conditions between proteins with and without a specific annotation. 17 317 unique UniProt protein IDs were identified. To minimize the impact of outliers, only annotations containing over 300 unique protein IDs are shown in this figure. For clearer visualization, log fold differences >3 are capped at 3, and those <−3 are capped at −3.

Our comprehensive search on the database, which catalogued *E. coli*‐expressed recombinant proteins, included entries corresponding to 17 317 unique UniProt IDs sourced from 19 799 research articles. This extensive dataset provides valuable insight into the commonly used induction conditions across a wide array of proteins. For instance, in Figure S4 (Supporting Information), we show that the most frequently used induction temperatures are 18, 20, and 16 °C, which accounted for 19.1%, 15.6%, and 13.6% of cases, respectively. The induction times are clustered predominantly around two ranges: 12–16 h (69.0%) and 3–6 h (14.3%), with isopropyl β‐D‐1‐thiogalactopyranoside (IPTG) concentrations commonly set to 1 millimolar or 0.5 millimolar. Additionally, as shown in **Figure** **5**, specific conditions are favored to maximize the expression of proteins with distinct annotations. For example, proteins associated with “disulfide bond,” “protease”, and “DNA‐binding” annotations tend to be expressed under shorter induction times (2–8 h) and higher temperatures (28–37 °C), whereas those related to the “cytoskeleton”, “cell cycle”, and “nucleus” are less commonly expressed under shorter induction times. Moreover, proteins that bind iron or whose functions are iron‐dependent are more frequently expressed at 28 °C for 24 h. Selecting such commonly used conditions would most likely enhance the folding, stability, and functionality of a protein with specific annotations, thereby making its expression more efficient and effective under these parameters.

### 2.8 Case Study 1: Analysis of Purification Strategies of Membrane Proteins

**Figure 6:**
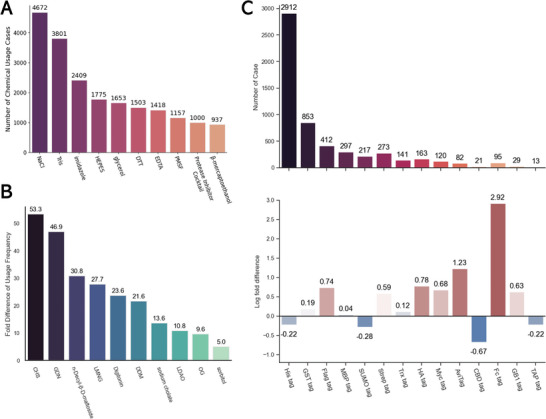
Statistics on Chemical Usage and Fusion Tags in Membrane Protein Purification. A) Top 10 most frequently used chemicals. B) Top 10 differences in chemical usage frequency between membrane proteins and non‐membrane proteins. 5252 unique membrane proteins and 17 581 unique non‐membrane proteins were identified by our article extraction tool. C) Instances of fusion tags on membrane proteins and the log fold differences in tag usage between membrane proteins and non‐membrane proteins.

Membrane proteins play crucial roles in biological processes, and their comprehensive characterization requires effective purification strategies. Purifying membrane proteins is challenging because they are typically present in low quantities and usually need detergents or stabilizers to be soluble in aqueous solutions. Our search identified 5252 distinct membrane proteins, each identified with a unique UniProt ID and categorized under the “membrane” cellular component, as annotated in the UniProt database. **Figure** **6**A illustrates the top ten chemicals used in the purification of membrane proteins. NaCl is the most frequently used, with 4672 instances, underscoring its importance in maintaining ionic strength and stability. Tris and HEPES, used in the purification of 3801 and 1775 different membrane proteins, respectively, are the two most popular buffer types. DTT (1503 instances) and ethylenediaminetetraacetic acid (EDTA, 1418 instances) are utilized for their reducing and chelating properties, respectively, which are crucial for maintaining protein stability and functionality.

Figure 6B presents the top ten variations in chemical usage frequency between membrane and non‐membrane proteins. Detergents show the most significant variations, with CHS (53.3‐fold), glyco‐diosgenin (GDN, 46.9‐fold), and DDM (30.8‐fold) being the most notable. This highlights the necessity of detergents in solubilizing membrane proteins from lipid bilayers, as detergents are amphipathic molecules that bind to membranes, lyse them, and form lipid/protein/detergent complexes.^[^ ^16^ ^]^ When initiating the purification of a new membrane protein, it is advisable to make the purification buffers with the most frequently used components. If the initial outcome is unsatisfactory, further optimization can be achieved by incorporating chemicals that are more frequently used in membrane protein purification. This strategy may reduce trial and error and increase the likelihood of successful purification.

The preference for certain fusion tags in the membrane protein purification process is highlighted in Figure 6C. The His tag, with 2912 instances, is the most commonly used tag. Small tags are often preferred because they minimally alter protein properties and improve the likelihood of successful expression in heterologous systems.^[^ ^16^ ^]^ Larger fusion tags, such as GST (853 cases), MBP (412 cases), and SUMO (217 cases), are also common, as they aid protein solubilization and folding. However, the frequency of these large fusion tags is not significantly higher among processes for the purification of membrane proteins as compared with non‐membrane proteins. Tags with relatively higher popularity in membrane protein purification include the Fc tag (18.48‐fold), Avi‐tag (3.44‐fold), HA tag (2.18‐fold), and FLAG tag (2.09‐fold). By focusing on these insights, researchers can streamline their purification processes, enhancing the efficiency and effectiveness of membrane protein characterization.

### 2.9 Case Study 2: Analysis of Detergents and Crosslinkers Usage in Preparing Protein Samples for Cryo‐EM Grids

**Figure 7:**
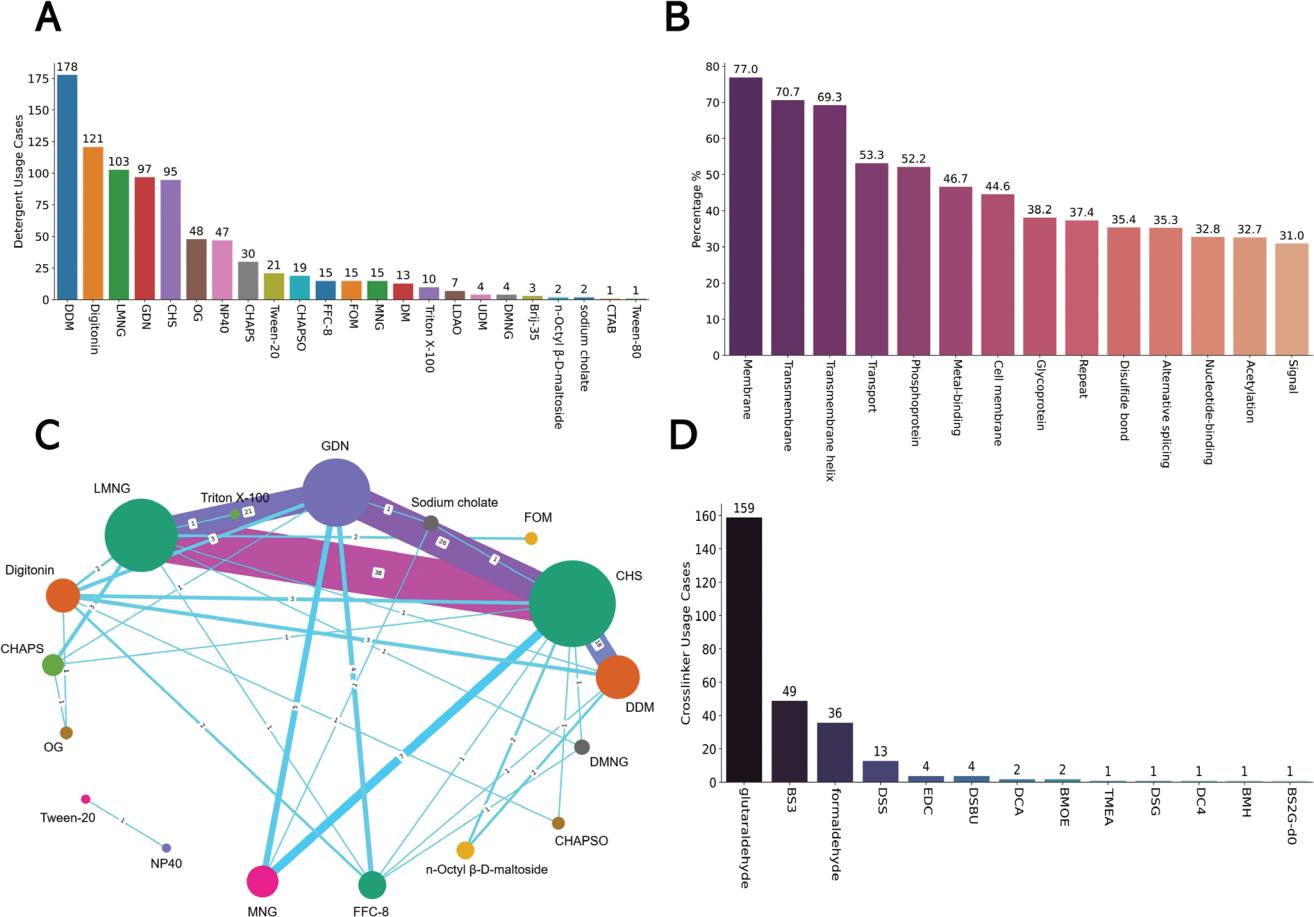
Statistics on Detergent and Crosslinker Usage in Preparing Protein Samples for Cryo‐EM Grids: A) Usage cases of detergents in cryo‐EM grid preparation from the 2746 screened articles associated with protein cryo‐EM structures. A certain detergent used for a unique protein was considered as a single case. B) Annotations of target proteins from unique PDB entries that involved detergent usage during cryo‐EM grid preparation. PDB entries of the same proteins from the same articles wtreated as a single case to remove duplication. Only annotations with a frequency >30% are displayed. C) Connection plot illustrating the co‐usage of detergent combinations in cryo‐EM grid preparation. The size of the nodes and the thickness of the connecting lines are proportional to the frequency of usage. We identified 97 articles that reported the use of detergent combinations from the 2746 screened articles. D) Usage cases of crosslinkers for stabilizing protein complexes prior to cryo‐EM grid preparation.

Detergents play a critical role in protein purification, particularly in the cryo‐electron microscopy (cryo‐EM) structure determination of membrane proteins. The amphiphilic nature of detergents allows them to interact with the hydrophobic domains of membrane proteins, enhancing solubility and aiding in the even distribution of protein molecules on the grid; it can also alleviate problems such as preferred orientation.^[^ ^17^ ^]^ To assess the prevalence of detergents usage in cryo‐EM sample preparation, we applied our Article Information Extraction Tool and identified 2746 articles that are associated with protein 3D structures in the RCSB Protein Data Bank. Of these, 1560 articles employed detergents during protein isolation, purification, or biochemical/biophysical assays, with 661 specifically reporting detergent usage during grid preparation for cryo‐EM experiments. **Figure** **7**A shows that the five most frequently used detergents in these studies are DDM (178 cases), digitonin (121 cases), LMNG (103 cases), GDN (97 cases), and CHS (95 cases). Notably, non‐ionic detergents—known for their mild denaturing effects and ability to disrupt protein‐lipid interactions without destabilizing the protein itself—dominate the usage. Among the PDB entries involving the use of detergents in grid preparation, 77.0% of the associated proteins or protein complexes were annotated as “membrane proteins”, as shown in Figure 7B. In addition, we identified other high‐frequency annotations, including “transport proteins” (53.3%), “phosphoproteins” (52.2%), and “metal‐binding proteins” (46.7%). When preparing cryo‐EM samples for proteins with these characteristics, the inclusion of detergents may improve grid quality and hence enhance the resolution of the structure. Additionally, 97 studies reported the use of detergent combinations, with the most common pairings being CHS‐LMNG (38 cases), CHS‐GDN (28 cases), GDN‐LMNG (21 cases), and CHS‐DDM (18 cases) (Figure 7C). However, in many cases, detergents were removed during the final stages of protein purification either through chromatography or dialysis, or by replacing them with nanodiscs or amphipols.

Besides detergents, chemical crosslinking emerges as another important strategy for stabilizing protein complexes for cryo‐EM experiments, particularly to mitigate structural heterogeneity. Through our analysis, we identified 241 articles that used chemical crosslinking prior to grid preparation (Figure 7D). Glutaraldehyde is the most frequently used crosslinker (159 cases), primarily through the GraFix (Gradient Fixation) approach, which involves ultracentrifugation of samples through a density gradient containing increasing concentrations of crosslinking reagent.^[^ ^18^ ^]^ Other commonly used crosslinkers includes bis(sulfosuccinimidyl)suberate (BS3, 49 cases) and disuccinimidyl suberate (DSS, 13 cases), which are typically employed to stabilize protein complexes, while formaldehyde (36 cases) is mainly used to stabilize protein‐DNA interactions. Collectively, these findings highlight the relative frequency of different types of detergents and crosslinkers in optimizing cryo‐EM sample preparation, contributing to more reliable and high‐resolution structure determination of protein complexes.

## 3 Discussion

Our efficient Article Information Extraction Tool is effective in extracting answers to questions related to protein characterization studies. As a notable flexibility of the tool, the embedding model and large language model can be replaced with other high‐performance models. By using higher‐ranking models, it is possible to achieve a lower error rate in extraction.^[^ ^19^ ^]^ In our study, to balance performance and processing costs, we determined that the combination of bge‐large‐en‐v1.5 and LLaMA 3 70B Instruct is currently the optimal choice.

**Figure 8:**
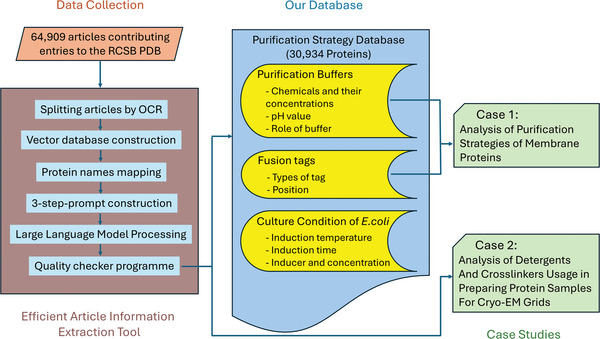
Overall schematic diagram of our Efficient Extraction Tool and the database. A total of 64 909 articles were processed using our Article Information Extraction Tool. Articles were segmented into chunks by Optical Character Recognition (OCR). The embeddings (1024‐dimensional vector) of the segments were built into the article vector database. The quality checker program examined the output of the LLM to ensure the quality and integrity of each entry. The tool resulted in the creation of a Purification Strategy Database containing data for 30 934 unique proteins. The database includes information on purification buffers, fusion tags, and *E. coli* culture conditions. Case Study 1 used data on purification buffers and fusion tags of membrane proteins from the database, while in Case Study 2, the extraction tool was applied directly to articles to analyze the usage of crosslinkers and detergents for the cryo‐EM sample preparation.

We applied our database to analyze fusion tags and chemical usage bias in membrane protein purification (**Figure** **8**). Additionally, beyond the three data categories of our database, the wide adaptive capabilities of large language models can potentially be applied to extract other protein‐related questions in the literature. To show its adaptability, we applied our extraction tool to articles associated with protein 3D structures solved by cryo‐EM, and successfully identified the frequency of different types of detergents and crosslinkers in optimizing cryo‐EM sample preparation.

By analyzing large‐scale data statistics, our approach can minimize trial‐and‐error in recombinant protein expression and purification, especially for the research focused on determining the protein 3D structures. For characterized proteins, sequence similarity searches may help researchers quickly identify the successful purification strategies from their homologs and orthologs. In particular, proteins in higher mammals often share a high degree of structural and functional similarity.^[^ ^20^ ^]^ As a result, purification strategies developed for a particular protein in one mammalian species (e.g., mice, cows, or pigs) can often be adapted for the use in other mammals (e.g., humans) with minor modifications. Furthermore, proteins can be classified based on function, structure, or physicochemical characteristics, and require different purification strategies tailored to each category.^[^ ^21^ ^]^ Therefore, if certain properties of the target protein have been characterized previously, methodological biases associated with specific annotations can guide the selection of appropriate purification strategies. In this study, we demonstrate biases in the selection of fusion tags, culture conditions in the *E. coli* expression system, and the use of chemicals in purification buffers.

Notably, in this study, we focus on the previous research contributing protein 3D structures to RCSB PDB. These studies not only obtained high‐quality protein samples essential for solving 3D structures, which require proteins with high purity and homogeneity, but also provided detailed purification procedures that can be reliably reproduced in other laboratories.^[^ ^22^ ^]^ Using our strategy, we found that each case of successful protein expression and purification corresponds to a specific combination of fusion tags, expression systems, culture and induction strategies, and purification buffers. However, in a single case, this combination can only be considered a viable option for obtaining a sufficient amount of pure and homogeneous protein for structural and functional studies. It cannot be deemed the optimal or sole choice. Furthermore, while an article may not provide a comprehensive description of all strategies for target protein expression and purification, it can confirm the compatibility of specific methods used for the target protein. For instance, the 3D structure of clathrin heavy chain (UniProt: P49951) was solved in 2003 (PDB: 1UTC), a company proprietary detergent was included in the lysis buffer (B‐PER Reagent) for protein isolation.^[^ ^23^ ^]^ From this, it can be inferred that proprietary detergent likely facilitates the purification of clathrin N‐terminal domain (a.a. 1–363). However, it cannot be concluded that obtaining clathrin N‐terminal domain without B‐PER Reagent is impossible; other detergents, such as Tween 20 or NP‐40, may also be effective. In fact, 13 years later, this protein with the same domain was successfully purified using Tween 20 for solving 3D structure (PDB: 5M5R) by another group.^[^ ^24^ ^]^ Therefore, each purification strategy in an article represents a sufficient but not necessarily the only condition for recombinant protein purification. Despite this limitation, the frequently selected strategies for proteins with similar properties or sequences can still help in choosing effective methods for a new protein with no prior purification record.

We have observed that different protein annotations exhibit distinct patterns in chemical usage during purification and choice of fusion tags. In future studies, our database search strategy could be utilized to train deep neural networks to predict protein purification strategies based on protein sequences and annotations. For instance, training a network to optimize buffer components can be approached as a multi‐label classification problem, with each component treated as a label. Given that each case is a sufficient but not necessary condition, training should be treated as an incomplete‐label supervised learning problem, which necessitates a more challenging model design and training process.^[^ ^25^ ^]^ By addressing these challenges, such models may offer a systematic and scalable approach to streamline protein purification workflows, reducing trial‐and‐error experimentation. Ultimately, the integration of machine learning with biological data holds significant potential to accelerate advancements in our understanding of proteins.

## 4 Conclusion

In conclusion, we developed an Article Information Extraction Tool optimized for articles associated with protein 3D structures. The design of the 2‐step‐LLM process and 3‐step‐prompt style significantly enhanced the accuracy of information extraction, which was demonstrated through the successful analysis of 64 909 articles associated with PDB protein 3D structure entries. Using the output from this tool, we constructed a comprehensive database of protein purification conditions, highlighting the distribution of buffer components, fusion tags, and *E. coli* expression conditions for obtaining high‐quality protein samples. Moreover, our correlation analyses revealed strategy biases in the purification approaches for proteins with varying properties. The robustness and flexibility of our tool were further validated through two case studies of method bias for membrane protein purification and crosslinker/detergent preferences for cryo‐EM sample preparation. These results collectively demonstrate that our extraction tool and database serve as a valuable resource for designing protein expression and purification protocols, offering practical insights to facilitate structural and functional studies.

## 5 Experimental Section

### Local Protein Information Database Construction

The local protein information database was served for the Efficient Article Information Extraction Tool, which was constructed from RCSB PDB database, UniProtKB/Swiss‐Prot, and UniProtKB/TrEMBL database. The PDB entry files in XML format were downloaded via FTP from the RCSB PDB website. Python package xml.etree.ElementTree was applied to extract the following information of each PDB entry: article title and doi, PDB entry title, protein sequences, expression system, method of solving the structure and the related UniProt accession ID. The UniProtKB/Swiss‐Prot and UniProtKB/TrEMBL databases were downloaded from ftp.uniprot.org. The Sample package was used to extract the following information of proteins with experimental 3D structure: recommended names, alternative names, gene names, keywords of protein annotations.

### Efficient Article Information Extraction Tool

To begin with, the PDF files of articles were downloaded from open source and preprocessed using the Partitioning Package of Unstructured library (v 0.12.0) in Python. By leveraging the Optical Character Recognition (OCR) functionality within this package, the articles were segmented into chunks containing no more than 2500 characters, with chunking based on titles. To build the vector database, the bge‐large‐en‐v1.5 model was employed to calculate the embeddings of each segment, storing them as 1024‐dimensional vectors. For each question of interest, the study created a template paragraph and computed the cosine similarities between its embedding and the embeddings in the article database. Segments were selected based on descending similarity scores and reconnected, and the total selected text per article did not exceed 12 000 characters.

The name mapping dictionary of protein full recommended names, alternative names, or abbreviations was constructed by using the webpages from the RCSB PDB and UniProt databases. This mapping information was processed by the Large Language Model (LLM) to determine the correct target protein names that appeared in each article. LLaMA 3 70B Instruct (4.00 bits, exl2 quantization version) and Mixtral 8×7B, a Sparse Mixture of Experts (SMoE) language model, were utilized for this task.^[^ ^26^ ^]^ Noted that the extraction for the final version of the dataset was performed using only the LLaMA 3 model. Subsequently, the identified protein names, questions of interest, and selected segments were used to construct prompts for the LLM. The output generated by the LLM was then examined by a quality checker program to enhance the accuracy of data extraction. This entire process was executed on a dual RTX 4090 GPUs workstation. The code for the tool was open‐sourced and included in the Supporting Information.

### Chemical Abbreviations and Synonyms Mapping

In practice, many articles use an abbreviation or synonym to represent a certain chemical. Additionally, OCR programs may introduce letter‐level typos for the chemical names. Therefore, a dictionary (provided in the source code folder of the tool) was constructed to correctly identify the names of chemicals. The score of matching was calculated by the following function.

(1) $$\text{Score}=\frac{\text{Lev}\left(a,b\right)}{\max\left(L_{a},\;L_{b}\right)}$$

where:

- Lev(*a*,*b*) was Levenshtein distance between two strings a and b, where a is the chemical name in the article, b is the candidates in the chemical mapping dictionary.
- *L_a_*, *L_b_* is the length of the strings a and b, respectively.

The candidate was selected with the following two criteria: the highest score of matching and its score equals or >0.85.

### Average Error Rate Calculation

The test dataset for the tool evaluation consisted of 50 articles, each detailing the buffer components used in the purification of 0–4 types of proteins. The following formula was used to calculate the average error rate of the data extraction.

(2) $$\mathrm{Average}\;\mathrm{Error}\;\mathrm{Rate}\;=\frac{1}{n}\sum_{i=1}^{n}\frac{FP_{i}+FN_{i}}{P+N}$$

where:

- *FP*_*i*_ is the number of false positive predictions for the i^th^ chemical.
- *FN*_*i*_ is the number of false negative predictions for the i^th^ chemical.
- *P* is the total number of positive samples in the dataset.
- *N* is the total number of negative samples in the dataset.

### Log Fold Difference Calculation

The following formula was used to calculate the log fold difference of a specific annotation in the database. The total number of unique UniProt IDs is 22 833 in the purification buffers dataset.

(3) $$\text{Fold Difference}=\ln\left(\frac{P_{1}/N_{1}}{P_{2}/N_{2}}\right)$$

where:

- *P*_1_ is the number of proteins with the specific annotation and used the chemical.
- *N*_1_ is the total number of proteins with the specific annotation.
- *P*_2_ is the number of proteins without the specific annotation and used the chemical.
- *N*_2_ is the total number of proteins without the specific annotation.

### Two‐Proportion Z‐Test

The following formula was used to calculate the Two‐Proportion Z‐Test of a specific annotation in the database of purification buffers containing 22 833 unique UniProt IDs.

(4) $$p=2\times \left|1-\Phi \left(\left|\frac{\frac{P_{1}}{N_{1}}-\frac{P_{2}}{N_{2}}}{\sqrt{\hat{p}\left(1-\hat{p}\right)\left(\frac{1}{N_{1}}+\frac{2}{N_{2}}\right)}}\right|\right)\right|$$

where:

- *p* is the *p*‐value of the Two‐Proportion Z‐Test.
- *P*_1_ is the number of proteins with the specific annotation for which the chemical was used in their purification.
- *N*_1_ is the total number of proteins with the specific annotation.
- *P*_2_ is the number of proteins without the specific annotation for which the chemical was used in their purification.
- *N*_2_ is the total number of proteins without the specific annotation.
- $\hat{p}$ is the pooled sample proportion, calculated as: $\hat{p}=\frac{P_{1}+P_{2}}{N_{1}+N_{2}}$
- Φ is the cumulative distribution function (CDF) of the standard normal distribution.

### Statistical Analysis

The heatmaps and correlation figures were generated by seaborn library (v 0.13.2). In statistical analysis, the Two‐proportion Z‐test was employed for two‐group comparisons. Log fold difference was applied to show the correlations of the groups with and without a certain annotation. Statistical analysis was performed using NumPy 1.26.2 in Python 3.10.13. *p* < 0.05 was considered statistically significant.

## Conflict of Interest

The authors declare no conflict of interest.

## Author Contributions

C.Z. and J.S. designed the research. C.Z. performed the research. C.Z. and J.S. analyzed and wrote the paper.

## Supporting information



Supporting Information

## Data Availability

The data that support the findings of this study are available from the corresponding author upon reasonable request.
