# Supplementary material for: Using Large Language Model to Optimize Protein Purification: Insights from Protein Structure Literature Associated with Protein Data Bank
Source: Adv Sci (Weinh). 2025 Feb 20;12(15):2413689. doi: 10.1002/advs.202413689 (PMC12005808; doi:10.1002/advs.202413689)
Supplement: Supplementary file 1 — Supporting Information [file ADVS-12-2413689-s001.docx]

Supporting Information

Using Large Language Model to Optimize Protein Purification: Insights from Protein Structure Literature Associated with Protein Data Bank

*Zhuojian Chen* and J Sivaraman**

**
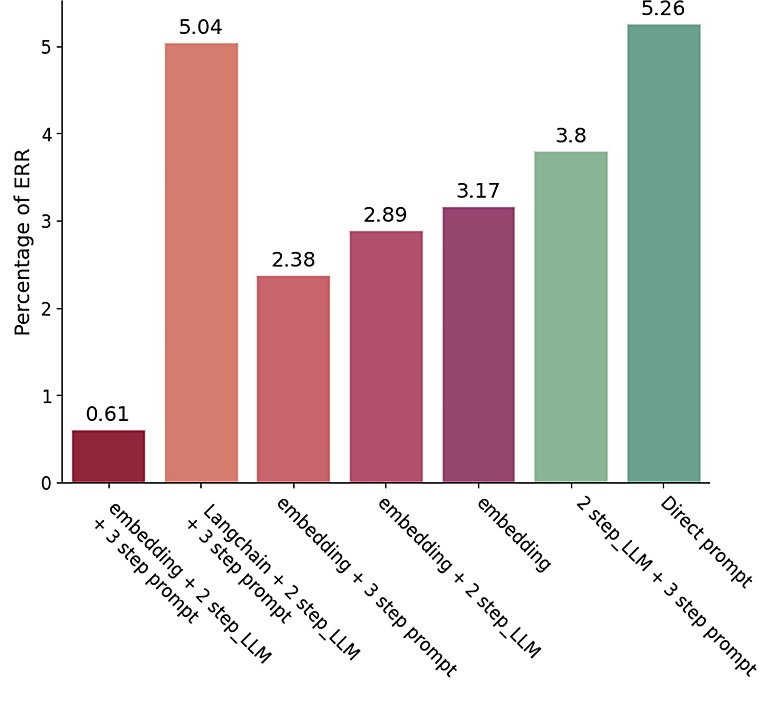
**

**Figure S1: Error rate of buffer component extraction from PDB linked articles:** The test dataset consisted of 50 articles, each detailing the buffer components used in the purification of 0 to 4 types of proteins, each associated with a unique UniProt ID.


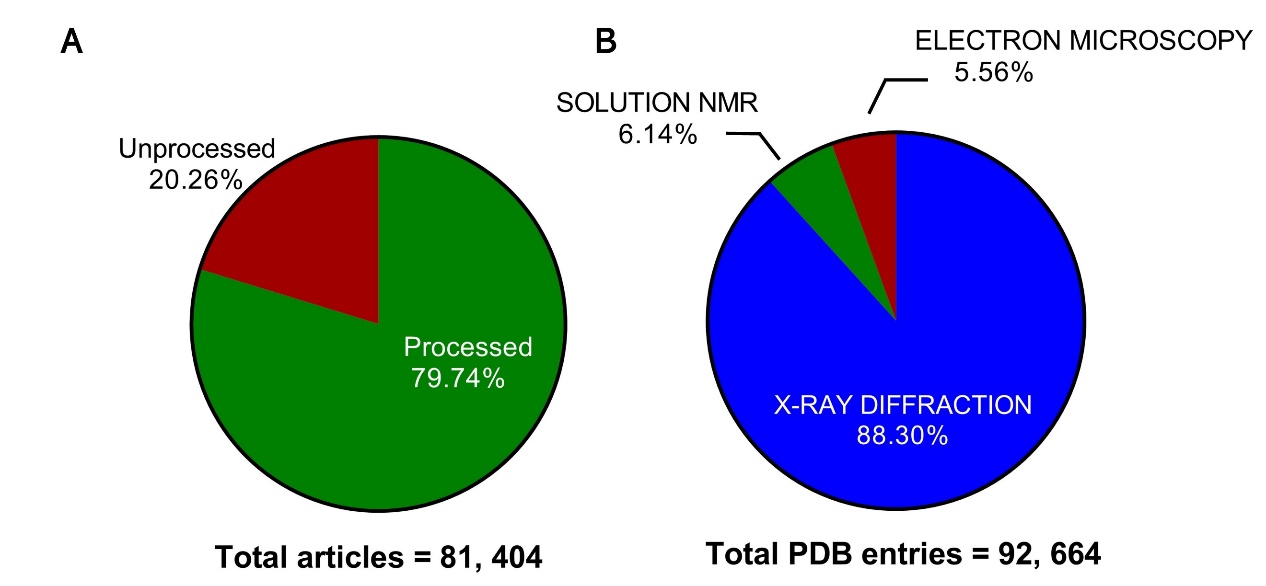


**Figure S2: Statistics of our database.** (A) Coverage of articles that contributed entries to the RCSB Protein Data Bank (updated in October 2024). (B) Distribution of methods used for determining protein 3D structures in our database.

**
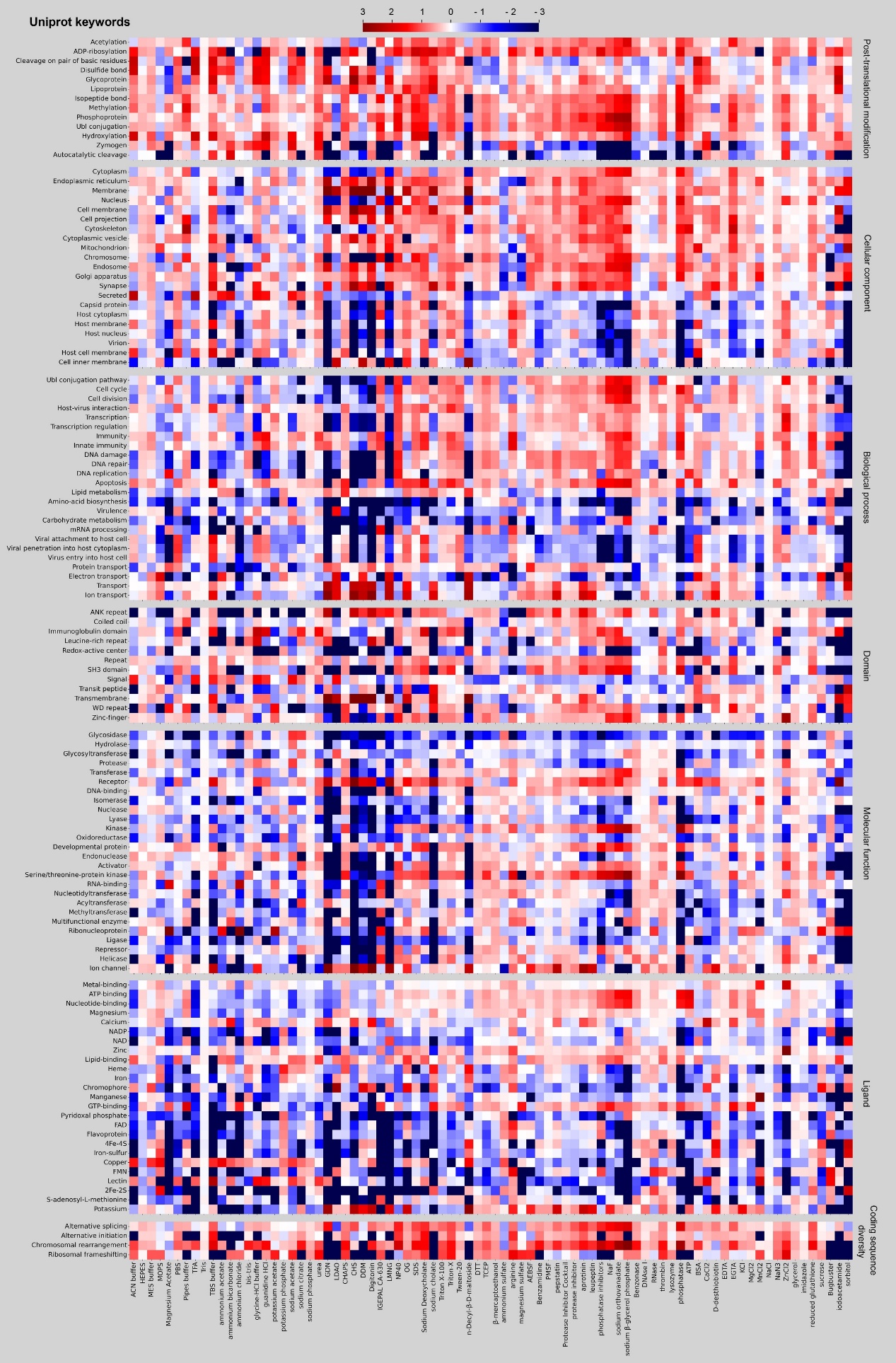
**

**Figure S3: Log fold-difference in buffer components between proteins with and without specific annotations**. The total number of unique UniProt IDs is 22,833 in this dataset. To minimize the impact of outliers, only annotations containing over 100 unique protein IDs in the categories “ligand,” “post-translational modification,” “coding sequence diversity,” and “domain” are included in our database. Additionally, annotations containing over 300 unique protein IDs in the categories “biological process,” “cellular component,” and “molecular function” are included. For clearer visualization, log fold differences greater than 3 are capped at 3, and those less than -3 are capped at -3.

**
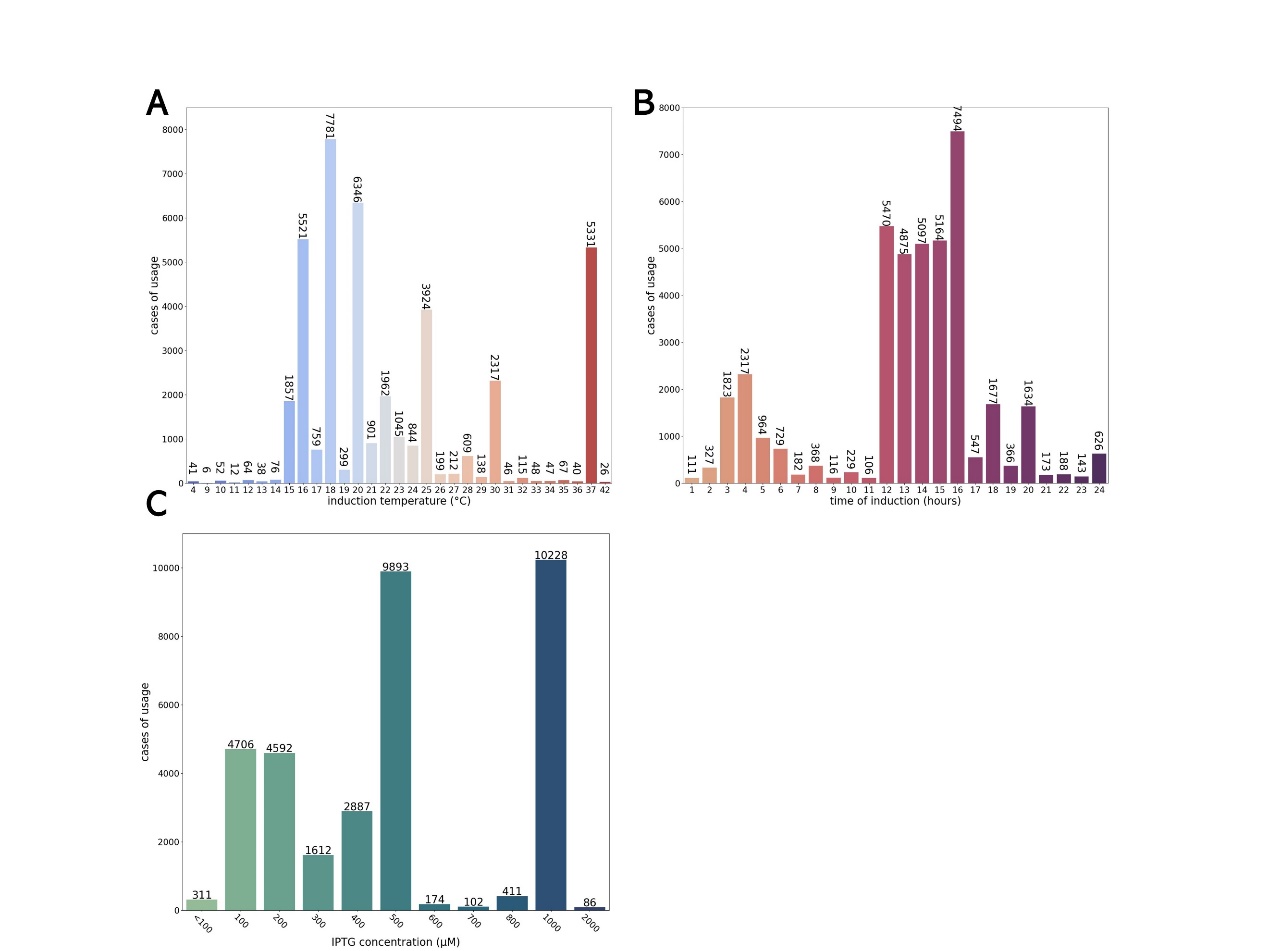
**

**Figure S4: Statistics of induction conditions of protein expression by *E. coli* in our database:** (A) Induction temperature. “Room temperature” is equivalent to 20~25 °C. (B) Time of incubation after the inducer added. “Overnight” is equivalent to 12~16 hours. (C) Concentration of IPTG. Only concentrations equal to or less than 2 millimolar are included.
